# Trial for the Prevention of Depression (TriPoD) in final-year secondary students: study protocol for a cluster randomised controlled trial

**DOI:** 10.1186/s13063-015-0929-1

**Published:** 2015-10-12

**Authors:** Yael Perry, Alison L. Calear, Andrew Mackinnon, Philip J. Batterham, Julio Licinio, Catherine King, Noel Thomsen, Jan Scott, Tara Donker, Sally Merry, Theresa Fleming, Karolina Stasiak, Aliza Werner-Seidler, Helen Christensen

**Affiliations:** Black Dog Institute, University of New South Wales, Hospital Road, Randwick, Sydney, NSW 2031 Australia; National Institute for Mental Health Research, The Australian National University, Canberra, Australia; Orygen, The National Centre of Excellence in Youth Mental Health, The University of Melbourne, Melbourne, Australia; South Australian Health and Medical Research Institute, Adelaide, Australia; Academic Psychiatry, Institute of Neuroscience, Newcastle University, Newcastle upon Tyne, UK; Department of Clinical Psychology, VU University, Amsterdam, The Netherlands; Department of Psychological Medicine, University of Auckland, Auckland, New Zealand

**Keywords:** Adolescents, CBT, Computerised CBT, Depression, eHealth, Prevention, RCT, School

## Abstract

**Background:**

Evidence suggests that current treatments cannot fully alleviate the burden of disease associated with depression but that prevention approaches offer a promising opportunity to further reduce this burden. Adolescence is a critical period in the development of mental illness, and final school examinations are a significant and nearly universal stressor that may act as a trigger for mental health difficulties such as depression. The aim of the present trial is to investigate the impact of SPARX-R, an online, gamified intervention based on cognitive behavioural principles, on the prevention of depression in secondary school students before their final examinations.

**Methods/Design:**

Government, independent and Catholic secondary schools in New South Wales, Australia, will be recruited to participate in the trial. All students enrolled in their final year of high school (year 12) in participating schools will be invited to participate. To account for possible attrition, the target sample size was set at 1600 participants across 30 schools. Participating schools will be cluster randomised at the school level to receive either SPARX-R or lifeSTYLE, an attention-controlled placebo comparator. The control intervention is an online program aimed at maintaining a healthy lifestyle. The primary outcome will be symptoms of depression, and secondary outcomes will include symptoms of anxiety, suicidal ideation and behaviours, stigma and academic performance. Additional measures of cost-effectiveness, as well as process variables (e.g., adherence, acceptability) and potential predictors of response to treatment, will be collected. Consenting parents will be invited to complete measures regarding their own mental health and expectations for their child. Assessments will be conducted pre- and post-intervention and at 6- and 18-month follow-up. Primary analyses will compare changes in levels of depressive symptomatology for the intervention group relative to the attention control condition using mixed-effects model repeated-measures analyses to account for clustering within schools.

**Discussion:**

This is the first trial of a universal depression prevention intervention delivered to school students in advance of a specific, significant stressor. If found to be effective, this program may offer schools a new approach to preparing students for their final year of schooling.

**Trial registration:**

Australian New Zealand Clinical Trials Registry identifier: ACTRN12614000316606. Registered 25 March 2014.

**Electronic supplementary material:**

The online version of this article (doi:10.1186/s13063-015-0929-1) contains supplementary material, which is available to authorized users.

## Background

By 2030, depression is predicted to be the second leading cause of disease burden worldwide [1]. Currently, in young people aged 10–24 years, it carries the greatest burden of disease [2]. Andrews et al. [3] calculated that only 13 % of this burden can be averted by available treatments and that, even with improved coverage, clinician competence and adherence, only 36 % of the burden of depression could potentially be alleviated using current knowledge and therapies. Indeed, Australian national data on service provision and access to treatment have demonstrated a considerable improvement over the previous two decades, with no discernible effect on prevalence, which, if anything, has increased [4]. This leaves the majority of the burden of major depression unaddressed. Further, relapse and recurrence of depression are frequent. More than 80 % of individuals with major depressive disorder (MDD) experience multiple episodes [5], with each additional episode predicting a greater likelihood of future recurrence [6], even after successful treatment [7, 8]. Given the potential chronicity of this disorder, intervening before the onset of a first episode represents a unique opportunity to change a lifelong trajectory of depression. A recent meta-analysis of 32 randomised controlled trials indicated a 21 % reduction in incidence of depression following the implementation of prevention programs [9]. Although there are some limitations to this analysis, prevention approaches represent a potential critical pathway to decreasing depression rates and the associated burden of this disorder.

Despite evidence indicating the overall effectiveness of prevention of depression [9], it remains unclear exactly which of the various prevention approaches is superior, for whom prevention programs are most effective, and under what circumstances the programs should be delivered. Universal prevention approaches are applied to whole populations, without regard to individual risk, whereas selective and indicated approaches target high-risk individuals on the basis of the presence of existing risk factors and subthreshold symptoms, respectively. Reviews in the literature have generally found larger effect sizes for targeted interventions. For example, data derived from a systematic review of school-based depression prevention programs found indicated programs to be the most effective [10]. Six of ten indicated trials demonstrated significant differences between the intervention and control conditions at post-test, with effect sizes ranging from 0.25 to 1.35. In contrast, only 9 of 23 universal interventions found significant effects over the control condition, though the range of effect sizes in these trials was similar to those in the indicated trials (*d* = 0.30–1.40). It should be noted, however, that it is easier to demonstrate success in indicated programs because individuals are selected on the basis of symptoms, meaning that there is greater opportunity for change (typically symptom reduction) to occur [11]. However, a key problem with the indicated approach in mental health is that individuals who are not yet symptomatic are not targeted in the intervention (see [12]). This means that individuals who may ultimately progress to develop symptoms will be missed. In contrast, by definition, universal prevention approaches provide greater scope and catchment of individuals who may be on the trajectory towards developing depression.

From an empirical perspective, whereas three recent universal prevention trials failed to find significant effects [13–15], a Cochrane review of 39 targeted (including both selective and indicated) and 31 universal psychological and educational prevention interventions for young people found evidence to support both targeted (number needed to treat [NNT] = 14) and universal programs (NNT = 8) [16]. With regard to diagnosis of depressive disorders, targeted interventions reduced diagnoses post-intervention (risk difference = −0.07) and at 3–9-month follow-up (risk difference = −0.06) relative to no intervention. Universal programs were also found to be effective immediately following the intervention and 3–9 months later (risk difference = −0.12 and −0.19, respectively). Similar results were found for depressive symptoms, with both targeted and universal interventions demonstrating evidence of efficacy post-intervention (standardised mean difference = −0.31 and −0.10, respectively) and at follow-up (standardised mean difference = −0.22 and −0.09, respectively).

Despite mixed evidence, the finding that universal programs may indeed be effective is important, given that they may also be easier to implement and are less stigmatising [17, 18]. Universal programs also have the added advantage of providing a foundation of skills upon which subsequent targeted programs can build [17]. Given that there is some support for universal programs but that this approach may not be effective in all circumstances, there is a clear case for further investigation of this kind of prevention. It is important, however, to determine the specific parameters within which universal prevention interventions are most likely to succeed. One potentially useful approach is to conduct universal interventions in advance of a stressor that is experienced universally and predictably.

Rates of depression begin to increase at 12–13 years of age, but the growth in incidence is continuous, with new cases of depression emerging at a similar rate throughout adolescence [18]. In determining the point at which a preventive intervention might be optimally delivered, it is notable that stressful life events act as predictors and candidate causal factors in the development of depression [19]. Final examinations represent a significant stressor for most adolescents. More specifically, in cultures where significant emphasis is placed on university entrance examinations in the final year of school, students tend to spend much more time doing schoolwork and less time in discretionary activities and to have more negative affective states across daily activities and higher rates of depression, relative to cultures without this focus on examinations [20]. Indeed, Kouzma and Kennedy [21] noted that the main sources of stress reported by final-year students in Australia were school-related. Amongst these stressors, examinations and academic outcomes were endorsed most strongly (see also [22–24]). Further, more than 40 % of year 12 students in Australia reported symptoms of depression, anxiety and stress that fall outside normal ranges [25]. In extreme cases, examination stress has even been linked to suicidal ideation, behaviour and completion [26, 27]. Because stress can lead to social and emotional difficulties, which can then interfere with academic achievement, programs that help students to manage stress before examinations may be beneficial, in terms of both improving mental health and enhancing academic performance [28].

To date, the potential for prevention has not been examined in the context of school-based stressors. However, researchers in one study examined the effect of a prevention program in a group of medical students in the United States [29]. Participants (mean age 27.6 years) were allocated to receive either MoodGYM, an online cognitive behavioural intervention, or an attention control program before their first-year internship. Compared with the control group, interns who completed the intervention were almost four times less likely to experience an episode of depression during their internship (13.0 % vs. 34.8 %). This finding suggests that prevention programs delivered before an anticipated period of increased stress may protect against the development of depression.

In terms of the most effective approaches to prevention interventions, one of the key factors to consider is the therapeutic orientation of the program. Hetrick and colleagues [30] recently conducted an exploratory meta-analysis, specifically examining program content, based on the trial data included in Merry et al.’s 2011 Cochrane review [16]. Of the 50 intervention arms examined, 38 were classified as based purely on cognitive behavioural therapy (CBT), 4 reported data on interpersonal psychotherapy (IPT) interventions and 8 were classified as ‘other’ in orientation. Results indicate not only that CBT is the most studied type of intervention for depression prevention but also that there is evidence to support its effectiveness in reducing the risk of developing a depressive disorder and reducing depressive symptoms both post-intervention and at follow-up. Mixed evidence was found for IPT, and other interventions were not found to be effective. The majority of programs in the review were delivered face-to-face to groups of adolescents in school settings, though the authors acknowledged that online delivery of programs is becoming increasingly popular.

With regard to setting, schools are an ideal location to deliver mental health interventions, not only because young people spend more time in these institutions than any other, but also because of the integral role schools play in students’ social, academic, cognitive, emotional and behavioural development [31]. Further, the provision of mental health interventions in schools (in contrast to providing students access to these interventions independently) necessarily increases uptake and adherence [32]. From cost-effectiveness, engagement and delivery perspectives, online technology is an advantageous means by which to deliver programs to students en masse [4]. Further, automated online programs guarantee fidelity because the information cannot be distorted or adapted during implementation of the program, nor does it rely on personal delivery [33]. These programs also have the benefit of avoiding a number of practical difficulties, such as the need for expensive face-to-face input from health professionals [34] or time-consuming teacher training (see, e.g., [14]). Therefore, delivering a universal, school-based CBT program to adolescents before a major stressor has the potential to prevent the onset of depression.

The present SPIRIT-compliant [35] protocol (see Table 1) describes the methodology for a cluster randomised controlled trial designed to test the effectiveness of SPARX-R, an online, CBT-based, gamified intervention designed to prevent the development of depression in students’ final year of secondary studies (known as the Higher School Certificate [HSC]). SPARX was designed as a treatment intervention for depression. For the purpose of the current study, SPARX will be revised for use as a preventive intervention, known as SPARX-R. SPARX has been shown to be non-inferior to treatment as usual (primarily individual face-to-face therapy) in depressed help-seeking adolescents [36], for whom use of the program resulted in clinically significant reductions in depressive symptoms. An online active control program (lifeSTYLE) focused on maintaining a healthy lifestyle will serve as an attention-controlled comparator. In addition to evaluating the relative impact on depressive symptoms, in the trial we will also explore the impact of SPARX-R on a range of secondary outcome variables, including academic performance, anxiety and suicidal ideation, as well as parental, process and cost-effectiveness variables.

**Table 1:** Items from the World Health Organisation dataset

Data category	Information
Primary registry and trial identifying number	Australian New Zealand Clinical Trials Registry identifier: ACTRN12614000316606
Date of registration in primary registry	25 March 2014
Secondary identifying number	U1111-1154-5917
Sources of monetary or material support	National Health and Medical Research Council
Primary sponsor	Black Dog Institute, University of New South Wales
Secondary sponsors	
Contact for public queries	YP, HC
Contact for scientific queries	YP, HC
Public title	Trial for the Prevention of Depression (TriPoD)
Scientific title	Inoculating final-year high school students: effectiveness of a universal prevention program for depression in adolescence
Country of recruitment	Australia
Health conditions or problems studied	Depression, anxiety and suicidal ideation and behaviour
Interventions	Active comparator: SPARX-R, an online, interactive game based on the principles of cognitive behavioural therapy designed to prevent depression in young people
	Placebo comparator: lifeSTYLE, an online program providing interactive content on a range of general health and well-being topics
Key inclusion and exclusion criteria	Inclusion criteria: male and female final-year secondary school students at participating schools
	Exclusion criteria: none
Study type	Study type: interventional
	Allocation: randomised
	Intervention model: parallel assignment
	Primary purpose: prevention
Date of first enrolment	February 2015
Target sample size	1600
Recruitment status	Recruiting
Primary outcome	Symptom levels of depression
Key secondary outcomes	Symptoms of anxiety, academic performance, suicidal ideation and behaviour, and stigma

A secondary aim of the trial is to determine the role of individual differences in response to both stress and the intervention, as these are likely to impact outcomes of interest. These variables (which may either increase vulnerability or act as protective factors) include family history of depression, social support [37], mastery [38], hopelessness [39], ruminative response style [40], neuroticism [41], belongingness and burdensomeness [42], disturbances in the sleep–wake cycle (see [43]), expressed emotion [44] and cortisol levels [45]. In addition to these factors, genetics are implicated in the onset of depression, both alone and in combination with environmental variables (see, e.g., [46]). Therefore, we also plan to examine a range of genetic markers to determine if, and in what way, genetics may interact with outcomes. Collection of cortisol and genetic data will be subject to approval of relevant governing ethics bodies.

### Hypotheses

With regard to the primary outcome measure, we predict that, relative to participants in the attention control condition, students who are assigned to the SPARX-R condition will demonstrate lower levels of depressive symptoms immediately following the intervention. We also anticipate that depressive symptoms for SPARX-R participants will be lower than those in the lifeSTYLE group at 6- and 18-month follow-up. For secondary outcomes, we expect that, relative to the lifeSTYLE group, participants in the SPARX-R group will perform significantly better in their HSC and demonstrate reduced anxiety, suicidal ideation and/or behaviour and stigma following the intervention and at follow-up. We anticipate that SPARX-R will have an effect on the parents of students allocated to this group, reducing their own symptom levels at 6 months, relative to parents of students in the attention control group. Finally, we predict that SPARX-R will be more cost-effective than lifeSTYLE, owing to fewer days out of role (e.g., studying, working), better quality of life and less use of health services at 6- and 18-month follow-up.

For those in the SPARX-R group, we predict that a positive response to the prevention program will be associated with the following variables at baseline: absence of family history of depression, high social support, high mastery, low rumination, low neuroticism and/or negative affect, low hopelessness, low thwarted belongingness, low burdensomeness, low sleep disturbance, unimpaired sleep–wake patterns, low expressed emotion, low cortisol levels and lack of genetic vulnerability factors.

## Methods/Design

### Trial design

This study is a cluster randomised controlled superiority trial with two parallel arms, consisting of an experimental condition and an attention-matched control condition. We will employ an adaptive design, such that accumulating data will be used to decide if or how to modify the study as it continues. To facilitate this design and to stagger recruitment, the trial will take place in two stages across consecutive years, with each stage beginning at the start of the academic calendar (February 2015 and February 2016). The intervention phase will last 5 weeks with a follow-up period of 18 months. Results from the 2015 cohort will be subject to an interim analysis to determine preliminary effects and determine whether any modifications are required in the conduct of the trial in 2016. Feasibility of continuation will also be assessed at this stage in terms of student and teacher acceptability of the intervention and any implementation issues that may arise during the first year of the trial.

### Ethical approval

This trial has received ethical approval from the New South Wales Department of Education and Training, the University of New South Wales, the University of Melbourne, Australian National University and Southern Adelaide Clinical Human Research Ethics Committees. Recruitment of schools will not be conducted until all local approval has been obtained. Any adverse events will be reported to the director of the Black Dog Institute and to the relevant human research ethics committees.

### Setting

The trial will be conducted in government, independent and Catholic secondary schools in New South Wales, Australia. Government schools in New South Wales are classified as either comprehensive or selective. Comprehensive schools accept enrolments of all students in the surrounding area, whereas selective high schools use an entry examination to enrol students with superior academic ability. The first stage of the trial will be conducted in government schools in metropolitan Sydney. Ethical approval to collect biological data in government schools was not granted. Therefore, measures of cortisol and genetic vulnerability will be collected only in the 2016 cohort (which we expect will comprise primarily independent and Catholic schools), pending ethical approval.

The 2015 cohort will comprise students from selective government secondary schools. Although study-related stress is a widely experienced phenomenon, academically gifted students may be particularly at risk of experiencing stress-related mental health difficulties during the final examination period, given the high rates of perfectionism in this group, as well as the pressure to succeed from parents, schools, the media and students themselves (see, e.g., [47]). Perfectionism has been shown to be a strong predictor of prospective stress in the context of an academically demanding end-of-semester period [48]. Further, the increased academic workload and considerable number of hours spent studying by students in a demanding education program is likely to compound this pressure and act as an additional source of stress [49]. Indeed, high-stakes examinations can lead to a range of negative outcomes in high-achieving students, including anxiety, depression, school absence, helplessness and ulcer [21, 50]. Thus, the characteristics of students in selective high schools make these schools a particularly relevant target for the present trial. It should be noted that some government schools have both selective and comprehensive streams. All students at these partially selective high schools will be eligible to participate; however, streaming status will be recorded, and school type will be accounted for in analyses.

### Participants

All adolescents enrolled in their final year of high school in participating schools will be invited to participate. The age range at baseline is 16–18 years. Owing to the universal nature of the study, there are no exclusion criteria. Students are free to receive additional support from health care professionals while participating in the trial, and this will be assessed and accounted for in secondary analyses.

### Recruitment

**Fig. 1:**
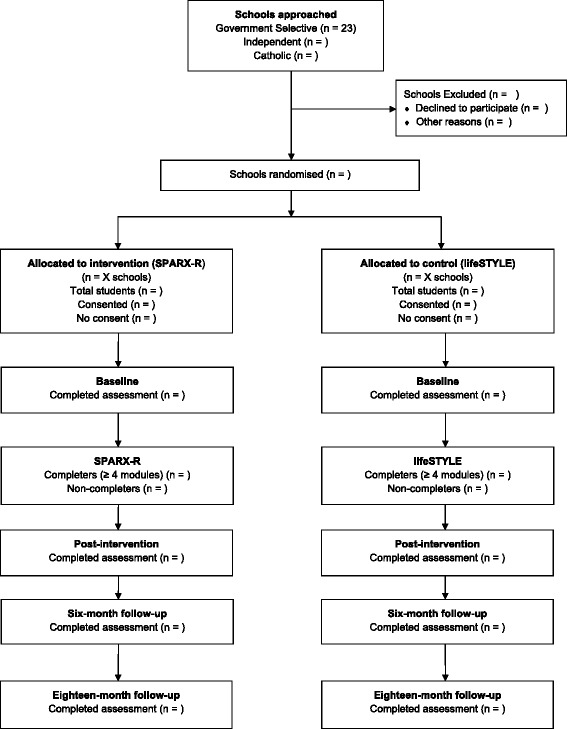
Planned flow of participants through the Trial for Prevention of Depression cluster randomised controlled trial

A flowchart outlining recruitment, randomisation and participation in this trial is provided in Fig. 1.

Thirty secondary schools across the state of New South Wales will be recruited to participate in the trial. School principals will be notified about the trial in writing and invited to allow their schools to participate. Research personnel may also meet with principals and/or teachers in person to provide information about the study and answer any questions. Once principals formally agree to their schools’ participating in the trial, parents and students will be informed about the trial.

Assignment to the intervention arm will occur at the school level. All students will complete the intervention, which will be integrated into the curriculum at each participating school. Consent will be sought from students and parents only with regard to the research component of the interventions (i.e., the completion of outcome measures and collection of other data). To maximise consent rates, information and consent forms will be distributed during the final term of the preceding school year. School administrators or class teachers will be provided with parental and student information and consent forms to pass on to all students enrolled in Year 12 the following academic year. Alternatively, schools may prefer to provide parents and students with consent forms at parent information sessions or to email parents the consent forms directly. Parents will be asked to either return a hard copy of the signed consent form to the school via their child or send a scanned or photographed copy via email. Parents will also be invited to participate in a parent survey by providing their email address and consent in an optional section of the consent form. Informed consent will be obtained from each participant, and students will be asked to return their signed consent forms to the school. In addition to participating in the study, students will have the option to provide consent for their school to provide their final examination results to the trial researchers and for the Trial for the Prevention of Depression (TriPoD) investigators to contact them in the future to inform them about any follow-up studies that may be conducted.

Using student email addresses provided by the schools, all students will be emailed a unique identification code before commencing the trial, which will allow them to register to access their allocated program via an online platform. This secure platform was designed specifically to facilitate the delivery of online interventions in a research context and can be configured to meet the needs of specific trials. For TriPoD, both of the interventions, as well as the research questionnaires, can be accessed through this platform. Those students without parental or personal consent will still register and access the program through the research platform; however, only those with consent will be given access to the research questionnaires as well. Students who do not have consent will complete an innocuous filler task while their peers are completing the research questionnaires.

### Sample size

Depression has an estimated annual prevalence of 8.3 % in adolescents [12], and between 8 % and 20 % of young people in community samples develop depression over an 18-month period [51–53]. On the basis of the age of the current sample, together with the timing of the intervention (before a major, universal stressor), we expect that onset rates will be high (up to 15 %). Calculations of required sample size for the primary outcome measure were based on detecting a post-intervention effect size of 0.20 in combined analyses of data collected from 2015 and planned 2016 cohorts. This estimate is based on previous depression prevention research using an online intervention [54]. Although the expected effect is small, it reflects the universal nature of this intervention. Power was set at 0.80, α = 0.05 (two-tailed) and a correlation of 0.5 assumed between baseline and endpoint scores. To allow for possible clustering effects (i.e., participants from the same school having characteristics and outcomes more alike than between schools), a design effect [55] was calculated assuming an intraclass correlation coefficient (ICC) of 0.02 and an average class size of 25. The estimate of the ICC was derived from a previous Australian school-based study that found a non-significant ICC of 0.02 [54]. The estimated sample size was 1166. The target size sample size was set at 1600, or 800 students per condition, to accommodate an attrition rate of approximately 20 % (see [56]). This translates to approximately 50 students (or 2 classes) per school in 30 schools. An interim analysis of students in the 2015 cohort will involve approximately 300 students per arm. Under the assumptions outlined above, there will be 80 % power to detect an effect size of 0.40 standard deviations between groups.

### Randomisation

Each participating school (cluster) will be randomised to complete the SPARX-R or lifeSTYLE program. School-level randomisation will be used to avoid contamination between conditions, for the ecological validity of providing the intervention at the cluster level and for practical convenience. A statistician not involved in the implementation of the trial will randomly allocate schools using the allocation sequence outlined below. The identity of schools will be concealed from the statistician during this process.

Randomisation in cluster randomised trials is problematic because the number of clusters involved is usually relatively small. In large, individually randomised trials, balance in the makeup of each arm is achieved as a function of the numbers involved. Balance between arms can be achieved by stratification or minimisation [57]. Stratification can accommodate only a limited number of balancing variables, and minimisation is based on incremental assignment of participants to groups. In TriPoD, the number of potentially relevant variables to be balanced between arms is quite large (e.g., sex, number of enrolled students, Index of Community Socio-Educational Advantage school index, language background other than English), and all available sites will be enumerated before commencement of the trial. Achieving approximately equal numbers of participants in each arm is also an aim of the allocation design, as this will determine the study’s power in the absence of substantial clustering effects. Accordingly, this attribute of each school will be used not to estimate the degree of balance, but rather to screen potential grouping after balanced combinations have been selected.

We will use the method developed and implemented by Carter and Hood [58] to ensure balance between intervention arms. This enumerates all possible divisions of the available schools into two groups and calculates a balance index [59] for each of these groupings. The 10 most balanced groupings will be retained after excluding any grouping resulting in a deviation of more than 10 % in predicted individual participant sample size between inventions. The allocation schedule will be selected at random from the groupings retained.

### Interventions

#### SPARX-R

The experimental intervention in this trial is SPARX-R, a revised version of SPARX, which was originally developed using an unguided, interactive fantasy game format that provides CBT skills to treat mild to moderate symptoms of depression in help-seeking adolescents. SPARX resulted in a decline in symptoms of depression and was shown to be non-inferior to treatment as usual, which consisted primarily of face-to-face therapy with a counsellor, general practitioner or clinical psychologist [36]. In this trial, response rates and treatment satisfaction did not differ between groups, nor did symptoms of depression, anxiety, hopelessness or quality of life, at post-intervention and 3-month follow-up. Further, remission rates were higher in the SPARX group. A pragmatic randomised controlled trial in a group of young people excluded from mainstream education also found SPARX to be superior to waitlist control with regard to reducing symptoms of depression [60]. Attitudes towards SPARX have been predominantly positive, with youth workers in New Zealand, young people alienated from mainstream schooling and rural Australian youth endorsing the program as a viable alternative to traditional therapeutic interventions [61–63]. Indeed, students in alternative education felt that SPARX should be offered to all adolescents in courses like their own, as they felt that providing the intervention at an indicated or targeted level might be stigmatising and that all could benefit from the lessons provided [64].

SPARX-R uses the same content as SPARX but is framed as a preventive intervention. For example, in the original version, participants are told that ‘*SPARX was made to help young people who feel down or depressed*’. In contrast, the SPARX-R introduction informs participants that ‘*this version of SPARX was made to help young people who are having hassles and feeling down, stressed or angry a lot of the time. Even if you are doing fine, SPARX-R can help strengthen your skills for dealing with problems when they do come along*’. Participants using SPARX-R choose and personalise an avatar and are led through the program by a virtual guide character who provides context and instruction and relates the content of the program to the participant’s real-life experiences. The participant navigates through a series of challenges and obstacles within a fantasy world that has been overrun by gloomy, negative, automatic thoughts. The student’s mission is to restore balance in the game world. The program has seven modules (levels), each of which takes approximately 20–30 minutes to complete. The modules cover the following topics: (1) finding hope, (2) being active, (3) dealing with strong emotions, (4) overcoming problems, (5) recognising unhelpful thoughts, (6) challenging unhelpful thoughts and (7) bringing it all together. SPARX-R will be delivered to participants via the internet on desktop computers in school classrooms.

#### lifeSTYLE

lifeSTYLE is an adaptation of an interactive, online program that was originally developed as a control intervention for a previous trial targeting adults with suicidal thoughts [65]. The format and structure of the program have been retained, but the content has been adapted to suit the needs of a younger group. The aim of the intervention is to provide an engaging and useful resource for young people that matches the intervention in terms of duration and attention but without providing any strategies or techniques to prevent or manage mental health difficulties. The program also includes seven modules (approximately 25 minutes each) and covers the following topics: (1) independence, (2) participating in your community, (3) work skills, (4) mobile phone safety and hygiene, (5) healthy skin, (6) sustainable eating and (7) maintaining a healthy home environment. Each module includes information about the specified topic as well as interactive activities such as quizzes, myth busters, videos and scenarios that students can reflect on and respond to. As with SPARX-R, the intervention is delivered to students in school classrooms via the online platform on desktop computers.

### Procedures

Immediately before the baseline assessment, during an allocated class period, school teachers will provide students with a URL that will take them to the trial registration page. Students will use their unique identification code to register at this page and set up a personal password. Students will also be required to provide a personal email address and mobile telephone number so that they can be contacted during the follow-up period after they have left school.

Students with parental and personal consent will complete questionnaires on three occasions during class time (pre-intervention, post-intervention and 6-month follow-up), as well as an additional 18-month follow-up questionnaire once they have graduated from secondary school. The timing of assessments was designed such that the 6-month follow-up will be conducted just before the trial HSC examination period, one of two major examination periods during the final year of school.

All assessments will be conducted via the online platform. Teachers will direct students to log on to the platform during the first three assessments, and participants will be emailed a link to log on directly from their personal computer on the final assessment occasion. All students will complete their allocated intervention (SPARX-R or lifeSTYLE) over the course of approximately 5–7 weeks in school via the online platform. Schools will timetable the intervention sessions to fit in with their respective schedules, but they will be advised to space sessions out so that students have sufficient time to reflect on the material presented in each module. On each assessment occasion, students who do not complete their assessments during the scheduled class time (owing to absence or other conflicting duties) will be asked to complete the questionnaires in their own time. A reminder email will be sent 1 week after the scheduled assessment, and a final reminder will be sent another week later to any student who still has not completed the scheduled assessment. Trial researchers will monitor completion of assessments and will remain in close contact with participating schools to ensure that school staff encourage assessment and intervention completion by all participating students. Students will no longer be able to access or complete assessments 1 month after their school’s scheduled assessment date.

### Measures

The administration schedule for each of the assessment measures described below is provided in Table 2.

**Table 2:** Assessment administration schedule

Assessment	Construct	Baseline	Post-intervention	6 months	18 months	Other
Primary outcome						
MDI	Depression symptoms	X	X	X	X	
Secondary outcomes						
ATAR	Academic performance					Collected via schools after final examination results are released
SCAS	Anxiety symptoms	X	X	X	X	
YRBS	Suicidal ideation	X	X	X	X	
DSS-P	Personal stigma	X	X	X	X	
Predictor variables						
SSSS	Social support	X				
PMS	Mastery	X	X	X		
RRS	Rumination	X	X	X		
PID-5-BF	Personality traits	X				
HPLS-Brief	Hopelessness	X				
INQ	Belongingness/burdensomeness	X				
ACSS	Capability	X				
ISI	Sleep disturbance	X				
MEQr	Sleep–wake patterns	X				
PS/PC	Expressed emotion	X				
Cost-effectiveness variables						
TiC-P	Service use			X	X	
Questionnaire	Days out of role			X	X	
AQoL	Quality of life			X	X	
Process variables						
Questionnaire	Expectation of success	X				
Intervention data	Adherence					Collected automatically via SPARX-R and lifeSTYLE
Questionnaire	Acceptability, usability		X			
IIAM	Reasons for dropout		X			
Parent outcomes						
PHQ-9	Depression	X		X		
GAD-7	Anxiety	X		X		

*ACSS* Acquired Capability for Suicide Scale; *AQoL* Assessment of Quality of Life; *ATAR* Australian Tertiary Admission Rank; *DSS-P* Depression Stigma Scale–Personal Stigma subscale; *GAD-7* Generalized Anxiety Disorder 7-item scale; *HPLS* Hopelessness Scale for Children; *IIAM* Internet Intervention Adherence Measure; *INQ* Interpersonal Needs Questionnaire; *ISI* Insomnia Severity Index; *MDI* Major Depression Inventory; *MEQr*  Reduced version of the Morningness Eveningness Questionnaire *PS/PC* Perceived Sensitivity/Perceived Criticism scale; *PHQ-9* Patient Health Questionnaire-9; *PID-5-BF* Personality Inventory for DSM-5; Brief Form; *PMS* Pearlin Mastery Scale; *RRS* Ruminative Response Scale; *SCAS* Spence Children’s Anxiety Scale; *SSSS* Schuster Social Support Scale; *TiC-P* Trimbos/iMTA questionnaire for Costs associated with Psychiatric illness; *YRBS* Youth Risk Behavior Survey

### Primary outcome measure

#### Major Depression Inventory

The Major Depression Inventory is a 12-item self-report measure of depressive symptoms [66]. The items of the MDI evaluate the presence and duration of depressive symptoms according to criteria of both the International Classification of Diseases, 10th revision (ICD-10), and the *Diagnostic and Statistical Manual of Mental Disorders, Fourth Edition* (DSM-IV) [67]. Participants rate the degree to which they have experienced each of the 10 symptoms over the preceding 2 weeks on a 6-point scale, ranging from 0 (at no time) to 5 (all of the time). The MDI has acceptable sensitivity and specificity for the diagnosis of depression according to the ICD-10 and DSM-IV [66]. To accord with DSM criteria and evaluate functional impairment, we included an extra question on the MDI asking participants to indicate (on the same scale) the degree to which their depressive symptoms interfered with activities of daily living over the previous 2-week period.

### Secondary outcome measures

#### Screen for lifetime presence of a major depressive episode or manic episode

Screening questions from the Structured Clinical Interview for DSM-IV disorders–Mood module [68] were included to provide an indication of a lifetime presence of a major depressive episode or manic episode. Mood, anhedonia, mania and irritability are assessed, and participants respond by answering ‘yes’ or ‘no’ to whether they have ever had prolonged experiences of each symptom (2 weeks for mood and anhedonia, 1 week for mania and irritability).

#### Australian Tertiary Admission Rank

Participants’ academic performance will be assessed by means of their estimated Australian Tertiary Admission Rank (ATAR) scores. With students’ consent, schools will provide researchers with students’ HSC results, from which an estimate of each student’s ATAR score will be calculated using a standard online ATAR calculator.

#### Spence Children’s Anxiety Scale

The Spence Children’s Anxiety Scale is a 44-item measure comprising 6 subscales. The scale was designed to measure the severity of children’s and adolescents’ anxiety symptoms based broadly on DSM-IV criteria for anxiety disorders [69]. Respondents rate the degree to which they experience each symptom on a 4-point frequency scale, ranging from 0 (never) to 3 (always). Only items on the Social Phobia and Generalised Anxiety subscales will be administered in the present study. The scale has demonstrated high internal consistency and satisfactory test-retest reliability [18]. It was also reported to show both convergent [70] and divergent [18] validity.

#### Youth Risk Behavior Survey

The Youth Risk Behavior Survey (YRBS) was designed to assess health risk behaviours among school students in years 9–12. For the purposes of the present study, the YRBS will be abbreviated to three items concerning suicidal thoughts, plans and attempts over the preceding month, for which participants will provide a yes-or-no answer. Endorsement of any of these items will trigger the trial’s participant risk management protocol (see below). Studies have shown that the suicidality items demonstrate both substantial reliability [71] and good convergent and divergent validity in high school samples [72].

#### Depression Stigma Scale

The Depression Stigma Scale (DSS) is an 18-item measure that assesses personal and perceived stigma towards depression. In the present study, only the nine-item personal stigma subscale will be used. These items require participants to rate how strongly they personally agree with a statement about depression (e.g., ‘people with depression are unpredictable’) on a 5-point Likert scale ranging from 0 (strongly disagree) to 4 (strongly agree). The sum of each of the items yields a total stigma score, where higher scores indicate greater stigma. The personal stigma subscale of the DSS has been shown to have moderate internal consistency in an adolescent sample [73, 74].

### Predictor variables

#### Schuster Social Support Scale

The Schuster Social Support Scale is a 15-item measure of social support used to examine an individual’s social relationships with others and the associated impact on their emotional functioning. Each item is rated on a 4-point scale from 0 (not at all) through to 3 (all the time). The scale has demonstrated acceptable reliability coefficients [75]. For the present study, 10 items from 2 subscales which measure support from friends and family will be used.

#### Pearlin Mastery Scale

The Pearlin Mastery Scale is a widely used instrument designed to measure participants’ perceived sense of mastery over life outcomes. Respondents are required to rate how strongly they agree or disagree with a list of seven statements on a scale from 1 (strongly disagree) to 4 (strongly agree). The total scores range from 7 to 28, with higher ratings indicative of a higher level of self-mastery, or control of the forces that affect their lives. The scale has shown good construct and predictive validity (see [76]) and good internal consistency [77].

#### Ruminative Response Scale–short version

The Ruminative Response Scale–short version is a 10-item, self-report questionnaire in which participants reflect upon the causes, meanings and consequences of their negative mood states. Participants are asked to rate how well the statements reflect what they generally do when feeling sad, blue or depressed on a 4-point Likert scale from 1 (almost never) to 4 (almost always). Higher scores indicate a greater degree of rumination about negative feelings and experiences. The short version has been shown to be a reliable measure of rumination that is less confounded by depression than the original version [78]. The scale has good construct validity [79] and convergent and discriminant validity [80]. It also has adequate internal consistency (α = 0.77 and 0.72 for the brooding and reflection subscales, respectively) and test-retest stability [78].

#### Personality Inventory for DSM-5 Brief

The Personality Inventory for DSM-5, Brief Form (PID-5-BF), is a 25-item self-rated personality trait assessment scale developed by the American Psychiatric Association’s Personality Disorders Work Group [81]. It measures the five proposed DSM-5 personality trait domains, including negative affect, detachment, antagonism, disinhibition and psychoticism, with each domain composed of five items. The scale asks respondents to rate how well the item describes themselves generally on a 4-point scale from 0 (very false or often false) to 3 (very true or often true). Overall scores range from 0 to 75, with higher scores indicating greater personality dysfunction. The scale has demonstrated good convergent validity with other widely used personality measures [82]. Further, though originally developed for adults, the PID-5 was also shown to be a reliable tool for measuring personality pathology within adolescent populations, with good reliability reported for personality trait domains and strong construct validity [83].

#### Hopelessness Scale for Children–Brief

The Hopelessness Scale for Children (HPLS)–Brief is a 5-item measure adapted from the HPLS [84]. Participants indicate their agreement with statements by circling ‘true’ or ‘false’, and responses are numerically scored. Higher scores reflect greater hopelessness or negative expectations about the future. Internal reliability and test-retest reliability were both found to be reasonable for the shortened version of the HPLS and comparable to the full 17-item version [85].

#### Interpersonal Needs Questionnaire

The shortened measure of the original 25-item Interpersonal Needs Questionnaire (INQ) contains 15 items designed to assess the extent to which participants feel like a burden on the people in their lives (i.e., perceived burdensomeness) and the extent to which they feel disconnected from others (i.e., thwarted belongingness). Statements are rated using a 7-point Likert scale in terms of how accurately they reflect participants’ recent feelings. After coding, higher scores reflect greater levels of thwarted belongingness and perceived burdensomeness. The scale was evaluated to have good internal consistency [86] and construct validity [87].

#### Acquired Capability for Suicide Scale

The Acquired Capability for Suicide Scale is a five-item self-report questionnaire that aims to measure an individual’s capability to enact lethal self-injury. Questions assess fear of death and the pain associated with dying. Items are rated on a scale from 0 (not at all like me) to 4 (very much like me), where higher scores are thought to be more indicative of one’s capacity for lethal self-injury. The scale has shown good convergent and discriminant validity [86] and high internal consistency in previous studies [88].

#### Insomnia Severity Index

The Insomnia Severity Index is a brief instrument with seven items that assess the nature, severity and impact of insomnia upon the responder. Questions assess both day- and night-time aspects of insomnia over the preceding 2 weeks and the associated interference with normal functioning. Items are rated using a 5-point Likert scale, from 0 (no problem) through to 4 (very severe problem). The scale has previously been shown to have excellent internal consistency [89] and good test-retest reliability, as well as face and content validity [90].

#### Reduced Morningness-Eveningness Questionnaire

The Reduced Morningness-Eveningness Questionnaire (MEQr) is a self-report measure consisting of 5 questions (reduced from the original version containing 19 items) designed to explore participants’ sleep and waking rhythms and habits. Known as a circadian questionnaire, the aim of the MEQr is to uncover the individual’s particular biological rhythm. The stability and external validity of the reduced questionnaire have been reported previously [91, 92].

#### Perceived Sensitivity/Perceived Criticism Scale

The Perceived Sensitivity/Perceived Criticism (PS/PC) scale is a very brief measure designed to rapidly assess levels of criticism from relatives as perceived by the participant [44]. The measure is designed to assess how critical the respondent perceives their relative to be and how sensitive the respondent is to this criticism. In the present study, we will ask specifically about how critical students perceive their mother and father to be and how sensitive they are to this criticism. The measure contains two single items, the first for perceived criticism and the second for perceived sensitivity to criticism, which are both rated on a scale from 1 (not at all critical/sensitive) to 10 (very critical/sensitive indeed). The PS/PC scale has been shown to reliably predict outcome for bipolar disorder [93].

### Cortisol and genetics

Cortisol data can be collected via hair strands in classrooms, and the procedure is non-invasive, painless, simple and quick. Saliva samples will also be collected in classrooms using standardised protocols. These data will be used for genetic analysis. Both types of biological data will be collected from students enrolled in the 2016 cohort, pending ethical approval.

### Cost-effectiveness outcomes

#### Trimbos/iMTA questionnaire for Costs associated with Psychiatric Illness

The Trimbos/iMTA questionnaire for Costs associated with Psychiatric Illness questionnaire was designed to assess the costs of psychiatric illness incurred within the health system, as well as the economic burden associated with production loss [94]. In the present study, participants will be asked to recall details about their consultations with health professionals that have occurred over the past 6 months, including clinic visits, appointments, telephone consultations and house calls. The self-report version of the questionnaire consists of 13 items, each requiring a yes-or-no answer and, if relevant, specification of the number of times each particular consultation occurred.

#### Days out of role

This brief questionnaire, administered at 6- and 18-month follow-up, consists of four questions assessing the number of days an individual is unable to attend to (or has some difficulty managing) normal activities (e.g., attending school, university or work) because of mental health problems. Questions were adapted from core items from the World Health Organization Disability Assessment Scale [95] to focus specifically on mental health.

#### Assessment of Quality of Life in four dimensions

The Assessment of Quality of Life in four dimensions (AQoL-4D) [96] is a standardised, self-report instrument designed to measure health-related quality of life across five domains: illness, independent living, social relationships, physical senses and psychological well-being. Each domain consists of three items; however, the illness subscale is not used for scoring purposes and therefore will not be collected during this trial. Some minor language adaptations were made to better suit the adolescent population (e.g., examples of household tasks were changed from ‘cleaning the house’ and ‘cooking’ to ‘cleaning your room’ and ‘helping with meals’). These adaptations were based on language in the AQoL-6D, a 20-item adolescent version of the same scale. The AQoL-4D has demonstrated sound psychometric properties, with reliability estimates ranging from 0.73 to 0.84 [97]. There is also some evidence to suggest that the AQoL is more sensitive than other health-related quality of life instruments to health states [98].

### Process variables

#### Expectation of success

At the pre-intervention assessment, participants will be asked a number of questions to determine their expectation of success associated with the intervention they are about to complete. These questions will assess (1) beliefs about internet treatments in general and (2) preference for trial condition. These questions are based on those used in previous research [99].

#### Acceptability of the intervention

At the post-intervention assessment, participants will be asked a range of questions designed to assess the acceptability and usability of the intervention they completed. These questions will measure how understandable and useful the program was, whether participants perceived that it affected their behaviour and whether they would recommend the program to others. Participants will also be provided with a list of techniques or skills specific to the program they completed (SPARX-R or lifeSTYLE) and will be asked which of these skills they have tried since completing the program and how helpful they were. These questions are also based on those used in previous research [99].

#### Adherence to the intervention

Participants’ adherence to their allocated intervention will be assessed using automated data collection through the SPARX-R and lifeSTYLE programs. For each participant, these data will include total number of visits to the site, average length of each visit and number of modules completed.

#### Reasons for non-adherence

Participants who are designated as non-completers of their allocated program will be asked to complete a brief questionnaire assessing their reasons for non-adherence to the program. These items are adapted from the Internet Intervention Adherence Measure [100], which was originally developed in the context of a paediatric encopresis intervention. The current items are similar to the adapted version of this measure developed by Farrer and colleagues [101] for use with an internet-based intervention for depression. Farrer et al.’s version of this measure includes two additional categories of reasons for non-adherence which may be relevant to the present trial: (1) engagement issues and (2) disease-specific issues (pertaining to depression).

### Parent outcomes

#### Patient Health Questionnaire-9

The Patient Health Questionnaire-9 (PHQ-9) is a self-administered nine-item depression screening and diagnostic tool based on DSM-IV criteria. The scale assesses the nine depression symptom criteria for frequency of occurrence during the previous 2 weeks, with scores ranging from 0 (not at all) to 3 (nearly every day). The psychometric quality of the PHQ-9 has been established [102].

#### Generalized Anxiety Disorder 7-item scale

The Generalized Anxiety Disorder 7-item scale is a widely used self-report anxiety scale which consists of seven items designed to measure the severity of symptoms of generalised anxiety disorder [103]. Participants rate individual statements on a 4-point frequency scale from 0 (not at all sure) to 3 (nearly every day) as they pertain to the previous 2 weeks. Individual item scores are summed to produce an overall score between 0 and 21, with higher scores indicative of greater anxiety. The scale was reported by its developers to have good reliability in addition to strong criterion, construct, factorial and procedural validity [103].

### Participant risk management protocol

All student participants will be given a contact sheet with the name and contact details of their school counsellor, as well as contact information for other counselling and support services (local and online). In addition, on each assessment occasion, participants’ responses will be monitored using an automated alert system that identifies severe mental health concerns, defined as endorsement of one or more items on the YRBS. Participants will be asked if they have experienced serious suicidal ideation or engaged in suicidal behaviour in the previous month. Endorsement of any item will trigger an alert whereby trial researchers will be notified immediately, prompting initiation of the study’s risk management protocol. The researcher will confidentially communicate the student’s name to the student’s school counsellor so that the counsellor can contact the student to offer immediate support. School counsellors of all participating schools will be briefed in advance about this risk management procedure. Students will also be provided with a popup alert via the online platform indicating that one or more of their responses suggest they may be distressed and encouraging them to seek support and/or use the contacts provided on their contact sheet. An email providing the same message will also be sent to their nominated email account.

### Data collection and management

All self-report data for the present trial will be collected via the online platform designed for and by the Black Dog Institute. Data collected routinely through the SPARX-R program will be passed to the online platform via an application programming interface. Data will be coded using a unique identification code. A list of emails associated with each identification code will be kept separately from the outcome database for the sole purpose of matching data to an individual participant in case of risk. Data will be stored securely on the university server, and only appropriate members of the TriPoD research team will have access to data collected in the context of this trial. All research personnel not involved with the day-to-day management of the trial will remain blinded to intervention allocation.

### Analysis

Primary analyses will be undertaken on an intention to treat basis, including all participants as randomised, regardless of treatment actually received. Effectiveness of SPARX-R will be established using a planned contrast of change from pre-test to the post-intervention in the active compared with placebo condition on the MDI within a mixed-effects model repeated-measures analysis [104]. School will be included in analyses as a random effect to evaluate and accommodate clustering effects. Variables used in determining allocation balance will be evaluated and retained in analyses where they are significant or quasi-significant. An unconstrained variance–covariance matrix will model within-individual dependencies. Transformation of scores, including categorisation, may be undertaken to meet distributional assumptions and accommodate outliers. Contrasts comparing change on the MDI from pre-test to other occasions of measurement will be undertaken as secondary analyses.

Models for binary outcomes analogous to the primary analysis approach will be used to compare the prevalence rates (MDD status) and other dichotomous outcomes between the two treatment arms at the endpoint and other occasions of measurement. Relative and absolute risk reduction, NNT (see [105]) and other relevant indices will be calculated for these outcomes and undertaken separately for participants who did not meet criteria for caseness at pre-test to assess the effect of the intervention on incidence. In analyses of scaled secondary variables, we will use methods comparable to those of the primary analysis. Subsidiary complier and related analyses will estimate the efficacy of SPARX-R in participants who completed modules from the SPARX-R program to produce a clinical impact (estimated to be four or more modules) [36]. In exploratory analyses, we will examine the effects of moderators and mediators of treatment (see [106]).

Interim analysis of outcomes of the 2015 cohort will be undertaken following the methods described above. Although the sample available will be of limited size, the outcome of this analysis will be used to guide the second stage of the trial, planned for 2016. Should there be clear evidence of lack of effect, based point estimates of effectiveness and associated confidence intervals, consideration may be given to abandoning second-stage recruitment or seeking possible modifications of the intervention.

### Dissemination

This trial is registered with the Australian New Zealand Clinical Trials Registry (ACTRN12614000316606). All protocol amendments will be listed on this registry. A lay-language summary of key findings will be provided to participating schools at the conclusion of the trial, and results will be disseminated to the scientific community via publications and conference presentations. This trial will be reported in accordance with the World Health Organisation Statement on Public Disclosure of Clinical Trial Results [107]. No restrictions have been imposed on the dissemination of the data by funders or any other party.

## Discussion

In the present trial, we will investigate the effectiveness of the SPARX-R intervention in the prevention of depression in young people. To our knowledge, this is the first trial of a universal prevention intervention delivered to school students in advance of a specific, significant exogenous stressor. Should it be shown to be effective, SPARX-R may offer an accessible, cost-effective, scalable and youth-friendly option for delivering a wide-scale prevention program in schools.

Although there is evidence to support the use of SPARX as a treatment for depression, its use as a preventive intervention has not yet been evaluated. Further, although previous qualitative studies have found the intervention to be feasible and acceptable in other samples (including students in alternative education and youth in rural Australia), the feasibility of this program in a school context and, in particular, with academically selective students is not known. In addition to acceptability information collected from students, staff feedback questionnaires will be provided to teachers who helped facilitate the trial to determine their perspectives on the feasibility of the intervention.

The focus on selective high schools in the first stage of this study has both advantages and limitations. In terms of advantages, the nature of the sample ensures that we are delivering the intervention to young people who may be particularly at risk of developing mental health difficulties caused by increased rates of stress, achievement orientation and perfectionism [47–49]. In contrast, this may limit the generalizability of our findings, as selective schools provide a unique sample of youth. Indeed, one may argue that the first year of the trial is in fact a universal intervention within a selective (high-risk) sample. Nevertheless, we intend to continue this study in non-selective schools following the initial stage of the trial, which will allow for comparison across different school types and students.

## Trial status

Twenty-three selective or partially selective high schools were approached to participate in the trial between August and October, 2014. Fourteen of the schools agreed to participate; however, four schools subsequently withdrew from the study. The final sample for the 2015 cohort consisted of two partially selective and eight selective government secondary schools. The 2015 cohort of schools completed their baseline assessments in February, 2015, followed by their respective online programs. All post-intervention assessments were completed by April, 2015 and six-month follow-up data was collected in August, 2015. Educational outcomes (HSC results) and 18 month follow-up data will be collected in 2016 for this cohort.

## Additional file

Additional file 1:
**Completed SPIRIT 2013 checklist of recommended items to address in a clinical trial protocol and related documents.** (DOC 121 kb)

## Abbreviations

ACSS: Acquired Capability for Suicide Scale
AQoL: Assessment of Quality of Life
ATAR: Australian Tertiary Admission Rank
CBT: Cognitive behavioural therapy
DSM-IV: *Diagnostic and Statistical Manual of Mental Disorders, Fourth Edition*
DSS-P: Depression Stigma Scale–Personal Stigma subscale
GAD-7: Generalized Anxiety Disorder 7-item scale
HPLS: Hopelessness Scale for Children
HSC: Higher School Certificate
ICC: Intraclass correlation coefficient
ICD-10: International Classification of Diseases, 10th revision
IIAM: Internet Intervention Adherence Measure
INQ: Interpersonal Needs Questionnaire
IPT: Interpersonal Psychotherapy
ISI: Insomnia Severity Index
MDD: Major Depressive Disorder
MDI: Major Depression Inventory
MEQr Reduced Morningness-Eveningness Questionnaire NNT: Number needed to treat
PS/PC: Perceived Sensitivity/Perceived Criticism scale
PHQ-9: Patient Health Questionnaire-9
PID-5-BF: Personality Inventory for DSM-5, Brief Form
PMS: Pearlin Mastery Scale
RRS: Ruminative Response Scale
SCAS: Spence Children’s Anxiety Scale
SSSS: Schuster Social Support Scale
TiC-P: Trimbos/iMTA questionnaire for Costs associated with Psychiatric illness
TriPoD: Trial for the Prevention of Depression
YRBS: Youth Risk Behavior Survey
